# Predictive Factors for Platelet Count Variation After Splenectomy in Non-Traumatic Diseases

**DOI:** 10.3390/jcm8010082

**Published:** 2019-01-12

**Authors:** Roxana M. Dragomir, Mircea D. Hogea, Marius A. Moga, Dana G. Festila, Calin P. Cobelschi

**Affiliations:** 1 Faculty of Medicine, Transilvania University, 500019 Brasov, Romania; mircea.hogea@unitbv.ro (M.D.H.); mogas@unitbv.ro (M.A.M.); 2 Faculty of Dental Medicine, Iuliu Hatieganu University of Medicine and Pharmacy, 400337 Cluj -Napoca, Romania; dana.festila@gmail.com

**Keywords:** splenectomy, non-traumatic diseases, multiple linear regression, prediction, platelet count

## Abstract

Background: The spleen plays a central role in a range of diseases. As such, great emphasis has been placed on the procedure of spleen removal, the benefits and the numerous associated complications. Given the immediate risk of the thrombotic complications, the aim of this study was to evaluate clinical and laboratory patient characteristics in non-traumatic diseases of the spleen, and to investigate possible predictive factors for platelet count variation following the procedure. Methods: A total of 72 patients who underwent splenectomy were included in this retrospective study. Correlation coefficients as well as multiple linear regressions were used to assess the relationship between post-splenectomy platelet count and various preoperative clinical and laboratory patient characteristics. Results: Following multiple linear regression analysis, we determined that 54.93% of post-splenectomy platelet count variation was explained by admission platelet count (*p* = 0.00), lymphocyte count (*p* = 0.04), WBC count (*p* = 0.00), LOS (*p* = 0.00), patient gender (*p* = 0.00), spleen accessibility on admission (*p* = 0.02) and PT (*p* = 0.00). Conclusions: Platelet count variation following splenectomy for non-traumatic diseases can be predicted by assessing preoperative patient characteristics. The implications of this study suggest that by means of a prediction model, patient care could benefit from assessing and addressing various preoperative factors that lead to these complications.

## 1. Introduction

While not being a vital organ in itself, the spleen plays a central role in a variety of diseases, given its special architecture and functions [1]. As a result, splenectomy is a surgical procedure performed as part of primary or second line treatment for a broad range of medical conditions, from traumatic injuries to hematological diseases and primary or secondary diseases of the spleen [1,2,3,4]. Based on the individual characteristics of each patient, the procedure of spleen removal can be performed via laparoscopic, classic surgery or by other, more advanced and minimally invasive techniques such as HALS, SILS, or robotic surgery [5,6,7,8].

The immunological, hematological and anatomical consequences of splenectomy have long been a source of interest and controversy among researchers and doctors alike [6,9]. Thus, the literature review offers a long array of studies on the complications following the procedure [10,11,12,13,14,15,16]. While attempts to investigate the predictive factors that lead to these complications have been made, we feel confident that enlarging the area of research with a more thorough statistical analysis could greatly benefit patient care [16,17,18,19,20].

Since the incidence of post-splenectomy reactive thrombocytosis is cited at 75–82% and the incidence of thrombosis associated with it is approximately 5%, depending on other associated risk factors, we centered our research on the hematological consequences of splenectomy [10,14].

Predicting postoperative platelet count might help with the decision of when and which patients could benefit from the procedure, as well as lower the risk associated with it.

Given the immediate risk posed by thrombotic complications associated with the procedure, the aim of this study was to establish predictive factors for platelet count variation in patients who underwent splenectomy for non-traumatic diseases. The proposed objectives are:

1. Post-splenectomy platelet count variation evaluation.
2. Development of a predictive model for platelet count variation using multiple linear regression analysis.

## 2. Material and Methods

The retrospective study was conducted based on the medical records of a sample of 72 patients who underwent either laparoscopic or open surgery splenectomy in two hospitals in Braşov County in Romania, during a 4-years period, between January 2013 and December 2016.

Considering the large variety of non-traumatic diseases of the spleen, the patients were grouped in 3 distinct categories: secondary malignancies of the spleen, hematological diseases with spleen involvement (hereditary spherocitocys, hemolytic anemia, thrombocytopenia) and primary diseases of the spleen (abscess, cyst and primary neoplasm).

Raw data was initially processed through Microsoft Excel software and further analyzed using STATA 12 software.

We conducted our research based on similar studies in the field, thereby applying similar methods and tests and including the same variables as well as new ones in our regression model. The included binary variables were gender, place of living, emergency admission, palpable spleen on admission, smoking, as well as the need for blood transfusion. Quantitative data was divided into admission and discharge values and included: age, weight, systolic and diastolic blood pressure, hemoglobin, hematocrit, RBC, WBC, PLT, MCV, MCH, MCHC, lymphocyte count, eosinophil count, basophil count, neutrophil count, ALT, AST, total bilirubin, urea, creatinine, glucose, Na, K, PT, INR and aPTT.

The onset of this study consisted of descriptive statistics, followed by Pearson correlation analysis. Furthermore, a 16 variables multiple linear regression analysis was also performed. The dependent variable was the discharge platelet count value. *p* value < 0.05 was used to infer statistical significance.

Heteroskedasticity was tested using the Breush-Pagan test. Multicollinearity was tested based on the analysis of the variance inflation factor.

One of the limitations of this study consisted in the lack of available data on spleen size and weight upon extraction. We tried to address this issue by including the accessibility of the spleen upon admission as a binary variable. Although it is a subjective evaluation of the spleen size, we are confident that a palpable spleen is easily distinguishable from a non-palpable one, to any medically trained personnel, and can therefore be used as a substitute.

As previously mentioned, the sample size consisted of 72 patients from 2 hospitals over a 4-years period. We ought to mention that these were not selected cases for our study, but rather all the cases that underwent splenectomy for non-traumatic diseases in the area. A more complex study can further be performed on a broader sample size, by enlarging the time frame, the area of research or by including the traumatic diseases of the spleen as well.

Another limitation, difficult to overcome, consisted in the inability to evaluate each patient following hospital discharge, due to lack of patient compliance. The inability of gathering data following hospital discharge, led to our study being conducted only on post-splenectomy platelet count values from the date of discharge.

## 3. Results

This research was performed on patients aged 27–88 with a mean age of 60.47 ± 12.7 years. Gender distribution revealed 39 female patients, and 33 male patients. Place of living analysis proved that 60 patients resided in urban areas and 12 patients lived in rural areas. Out of the 7 patients who passed away during their hospital stay, 6 were initially admitted to the ER. The length of hospital stays ranged between 1 and 108 days with a mean of 12.5 ± 12 days. Longer inpatient care was required for secondary malignancies of the spleen, followed by hematological diseases with spleen involvement and primary diseases of the spleen. Based on the medical records, only 25% of the patients had a palpable spleen on admission.

In order to determine if the three categories of diseases: hematological diseases with spleen involvement (*n* = 10), primary diseases of the spleen (*n* = 13) and secondary malignancies (*n* = 49) had any impact on the PLT count on discharge, a Kruskal-Wallis test was performed. The results revealed that there was no statistically significant difference in the PLT count between the three groups (Chi^2^(2) = 1.908, *p* = 0.3851).The Pearson coefficient analysis proved a positive correlation between admission and discharge platelet count (*r* = 0.4755, *p* = 0.000), no significant correlation was found to exist between patient age and discharge platelet count (*r* = 0.067, *p* = 0.5758), a positive correlation was also found between number of days in the hospital and the discharge platelet count (*r* = 0.3583, *p* = 0.002), similarly between WBC count and PLT count (*r* = 0.3747, *p* = 0.0012), as well as PT and PLT count (*r* = 0.2941, *p* = 0.0142) no significant correlation was found between PLT count and lymphocyte count, AST, ALT, bilirubin and PT.

The initial number of variables was 45, given that admission and discharge values were separated for the same variable. A total number of 16 variables were included in the initial multiple linear regression analysis with the aim of determining the relative contribution of preoperative patient characteristics to post-splenectomy PLT variation. Based on statistical significance (*p* < 0.05) and lack of multicollinearity (VIF < 10) criteria, 9 of the variables were excluded from the analysis. Thus, a restricted multiple regression was further performed (Table 1).

**Figure 1:**
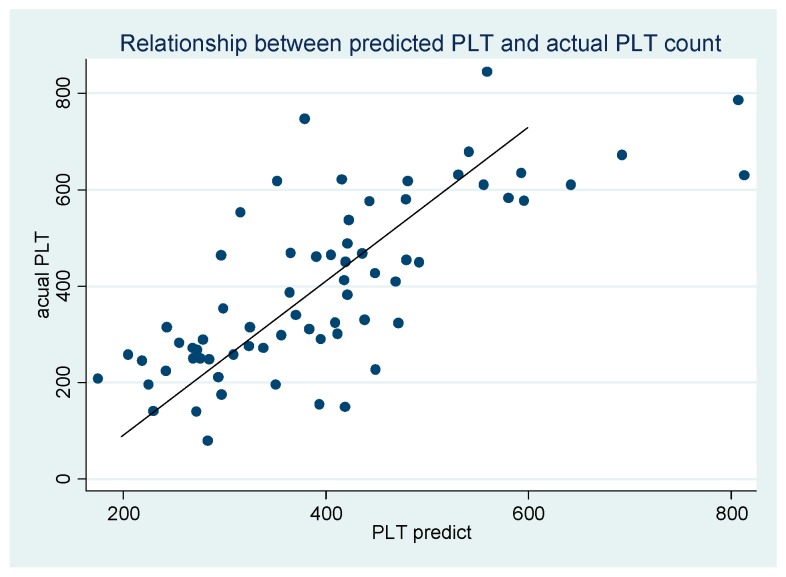
Relationship between predicted PLT and actual PLT. PLT_discharge_ = 16.07 + 0.48 PLT_admission_ + 92.6 Gender_male_ + 3.66 LOS + 92.31 Spleen_access_ + 8.85 WBC − 44.86 Lymph + 9.85 PT.

Predicted PLT was determined based on the regression equation. Its relationship with the actual PLT is presented in Figure 1, demonstrating a directly proportional correlation.

Heteroskedasticity is a major concern when applying a regression analysis, as the linear regression assumes that the variance of errors is constant across observations. The presence of multicollinearity in a multiple regression implies that independent variables in a regression model are correlated. Hence, the regression model was further diagnosed for lack of heteroskedasticity and multicollinearity. When testing for heteroskedasticity, the Breusch Pagan test produced a chi^2^ with 7 degrees of freedom of 4.12 with a *p* value of 0.7664, determining that there is no heteroskedasticity. Similarly, testing for variance inflation factor (VIF) < 10 and Tolerance (1/VIF) > 1/10 proved the lack of multicollinearity, as shown in Table 2.

## 4. Discussion

The aim of this paper was to identify possible factors that might influence the PLT count variation following splenectomy in non-traumatic diseases. Based on our research, the results show that 54.93% of the PLT variation is explained by this linear model, compared to 33.6% in other studies [17]. This notable difference can be attributed to a larger number of patients in our study, compared to other sources, as well as an increased number of independent variables, pertaining to this regression model.

Among the included variables, PLT preoperative values (*p* = 0.00), patient gender (*p* = 0.00), length of hospital stay (*p* = 0.00), spleen accessibility upon admission (*p* = 0.02), WBC count (*p* = 0.00) and PT (*p* = 0.00) proved a positive independent correlation, whereas lymphocyte count (*p* = 0.04) showed a negative correlation with postoperative PLT. Similar to our findings, other studies have shown PLT values post-splenectomy to be influenced by PLT preoperative values, spleen weight and lymphocyte count [17]. As suggested in the literature review, the reason for the negative correlation between lymphocyte count and platelet count is unclear.

The results suggest that for every one unit increase in PLT count on admission, PLT count on discharge will increase by 0.4854. *Ceteris paribus*, the patient with one more unit in PLT count on admission will also have a 0.48 increase in PLT count on discharge.

A more subtle and careful interpretation is needed in the case of LOS, as the raw data would simply imply that the higher the number of LOS, the higher the PLT count. LOS correlation with PLT count should be analyzed based on the necessary hospital days, meaning the degree of illness as well as the underlying pathology, type of splenectomy and other forms of organ dysfunction. This should avoid the error of implying that an earlier discharge would contribute to a lower PLT count and thus benefit the patient.

Our findings show that a higher accessibility of the spleen on admission determines a higher PLT count upon discharge. A higher PLT count is also inferred for male patients in comparison to female patients, following spleen removal. WBC count on admission showed a positive correlation with PLT count on discharge. A difference of a single second in PT between two patients should generate a 9.85 unit increase in PLT count on discharge.

In conclusion, similar to other studies, our data shows that a consensus regarding PLT post-splenectomy prediction could be reached by assessing and addressing various patient characteristics. Our research confirmed the results of other studies and revealed four other possible factors to be taken into account in such a prevision model, namely: patient gender, LOS, WBC, PT.

## 5. Conclusions

Most of the previous studies in the field were centered on a specific disease with spleen involvement and only one of the multiple types of techniques, mainly the laparoscopic approach. Our study encompassed various types of spleen diseases. Furthermore, it included both the classical and the laparoscopic approach of the procedure, in an attempt to reveal what are the possible predictive factors that have the ability to influence the PLT variation post-splenectomy, regardless of the disease.

Data analysis in medical research should be carefully interpreted, as mathematical findings do not always fall between clearly defined boundaries. As previously shown in respect to LOS and PLT count correlation, the individual context of the patient as well as medical reasoning must be taken into account. There was a significant variability in the number of hospital days, from one day to over one hundred days. Although the research on our sample of patients proved that LOS can be used as an independent variable, we believe that it should be further investigated if LOS can also function as a confounder for the underlying pathology, type of splenectomy, and other forms of organ dysfunction. The analysis of existing correlations between various clinical and laboratory patient characteristics, led to a predictive model that explains 54.93% of PLT count variation on discharge, thereby proving that previously unaccounted variables such as patient gender, LOS, WBC and PT should be also taken into consideration when assessing the potential risk of thrombotic complications. This result implies that there may be still many other patient characteristics that should be further analyzed. Our future goal is to reveal such variables in a cross-sectional study on a longer period of time and a broader number of patients from possibly other hospitals. We estimate that the coefficient of determination might be further increased by expanding the research to broader areas, an extended time frame and the inclusion of traumatic diseases as well.

It is worthwhile mentioning that in most cases, the procedure of spleen removal was performed 1–3 days after admission. Based on our findings, we conclude that a thorough preoperative assessment of the risk factors and clinical and laboratory variables will decrease the risk associated with the procedure, as well as improve the postoperative laboratory values and thus lead to fewer complications and an increased survival rate.

## Figures and Tables

**Table 1:** Restricted multiple linear regression.

Variable	Coefficient	*p*-Value
Platelet (×10^4^ µL^−1^)	0.4854659	0.000
Patient gender (Male, Female)	92.60927	0.006
LOS (days)	3.667707	0.004
Spleen accessibility (Y/N)	92.3197	0.020
Leukocytes (µL^−1^)	8.851022	0.006
Lymphocytes (µL^−1^)	−44.86827	0.048
PT (seconds)	9.855451	0.0048
Intercept	16.07465	
*N*	72	
*F* (7,60)	10.45	
Prob > *F*	0.0000	
*R* ^2^	54.93%	

**Table 2:** Multicollinearity test.

Variable	VIF	1/VIF
x_9_	1.16	0.862018
x_15_	1.15	0.870800
x_7_	1.13	0.886914
x_1_	1.11	0.900033
x_10_	1.10	0.909121
x_3_	1.07	0.934090
x_5_	1.06	0.939652
Mean VIF	1.11	

VIF: testing for variance inflation factor.

## Abbreviations

ALT	Alanine aminotransferase
AST	Aspartate aminotransferase
HALS	Hand-assisted laparoscopic splenectomy
LOS	Length of hospital stay
MCH	Mean corpuscular hemoglobin
MCHC	Mean corpuscular hemoglobin concentration
PT	Prothrombin time
RBC	Red Blood cells
SILS	Single-incision laparoscopic splenectomy
WBC	White blood cells
